# 5-(Biphenyl-4-yl)-3-(3-meth­oxy­benzyl­idene)furan-2(3*H*)-one

**DOI:** 10.1107/S1600536811031588

**Published:** 2011-08-11

**Authors:** Jerry P. Jasinski, James A. Golen, A. S. Dayananda, H. S. Yathirajan, Ravinesh Mishra

**Affiliations:** a Department of Chemistry, Keene State College, 229 Main Street, Keene, NH 03435-2001, USA; b Department of Studies in Chemistry, University of Mysore, Manasagangotri, Mysore 570 006, India; c Department of Pharmaceutical Chemistry, Faculty of Pharmacy, Jamia Hamdard (Hamdard University), Hamdard Nagar, New Delhi 110 062, India

## Abstract

In the title compound, C_24_H_18_O_3_, the dihedral angles between the mean planes of the five-membered furan ring and the meth­oxy-substituted benzene and the adjacent and outer biphenyl benzene rings are 2.43 (7), 4.48 (7) and 30.47 (8)°, respectively. The crystal packing is stabilized by weak C—H⋯O and C—H⋯π inter­molecular hydrogen bonds and π–π stacking inter­actions [centroid–centroid distances = 3.8752 (8) and 3.8331 (8) Å].

## Related literature

For potential anti-ulcer agents containing a furan­one structure, see: Felman *et al.* (1992 ▶). For the role of furan­ones in the biochemical processes of the human body, see: Rappai *et al.* (2009 ▶). For the gastrointestinal toxicity of acidic non-steroidal anti-inflammatory drugs (NSAIDs), see: Husain *et al.* (2010 ▶). For gastrointestinal side effects of NSAIDS, see: Cioli *et al.* (1979 ▶). For biologically active five-membered heterocyles such as butenolides and pyrrolo­nes, see: Husain *et al.* (2005 ▶); Khan & Husain (2002 ▶). For oxadiazo­les and triazoles, see: Husain & Ajmal (2009 ▶); Hashem *et al.* (2007 ▶). For a related structure, see: Burke *et al.* (2000 ▶). For bond-length data, see: Allen *et al.* (1987 ▶).
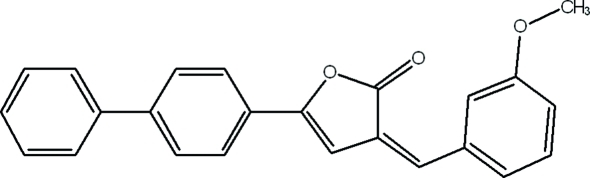


## Experimental

### 

#### Crystal data

C_24_H_18_O_3_
*M* *_r_* = 354.38
Monoclinic, 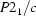

*a* = 19.8766 (8) Å
*b* = 6.9914 (3) Å
*c* = 13.2603 (6) Å
β = 107.735 (4)°
*V* = 1755.15 (13) Å^3^
*Z* = 4
Mo *K*α radiation
μ = 0.09 mm^−1^
*T* = 170 K
0.22 × 0.22 × 0.12 mm

#### Data collection

Oxford Diffraction Xcalibur Eos Gemini diffractometer
Absorption correction: multi-scan (*CrysAlis RED*; Oxford Diffraction, 2010 ▶) *T*_min_ = 0.981, *T*_max_ = 0.990
19756 measured reflections
4534 independent reflections
3310 reflections with *I* > 2σ(*I*)
*R*_int_ = 0.029

#### Refinement

*R*[*F* ^2^ > 2σ(*F* ^2^)] = 0.048
*wR*(*F* ^2^) = 0.143
*S* = 1.01
4534 reflections
245 parameters
H-atom parameters constrained
Δρ_max_ = 0.23 e Å^−3^
Δρ_min_ = −0.16 e Å^−3^

### 

Data collection: *CrysAlis PRO* (Oxford Diffraction, 2010 ▶); cell refinement: *CrysAlis PRO*; data reduction: *CrysAlis RED* (Oxford Diffraction, 2010 ▶); program(s) used to solve structure: *SHELXS97* (Sheldrick, 2008 ▶); program(s) used to refine structure: *SHELXL97* (Sheldrick, 2008 ▶); molecular graphics: *SHELXTL* (Sheldrick, 2008 ▶); software used to prepare material for publication: *SHELXTL*.

## Supplementary Material

Crystal structure: contains datablock(s) global, I. DOI: 10.1107/S1600536811031588/ci5197sup1.cif

Structure factors: contains datablock(s) I. DOI: 10.1107/S1600536811031588/ci5197Isup2.hkl

Supplementary material file. DOI: 10.1107/S1600536811031588/ci5197Isup3.cml

Additional supplementary materials:  crystallographic information; 3D view; checkCIF report

## Figures and Tables

**Table 1:** Hydrogen-bond geometry (Å, °) *Cg*1 is the centroid of the C1–C6 ring.

*D*—H⋯*A*	*D*—H	H⋯*A*	*D*⋯*A*	*D*—H⋯*A*
C2—H2*A*⋯O3^i^	0.95	2.56	3.3358 (18)	138
C5—H5*A*⋯*Cg*1^ii^	0.95	2.69	3.4762 (16)	141

Symmetry codes: (i) 
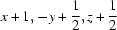
; (ii) 
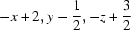
.

**Fig. 1.**
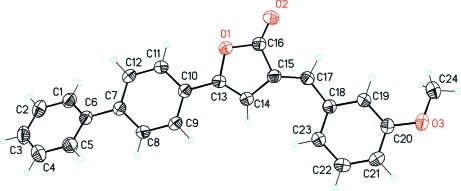
Molecular structure of the title compound, showing the atom-labeling scheme and 50% probability displacement ellipsoids.

**Fig. 2.**
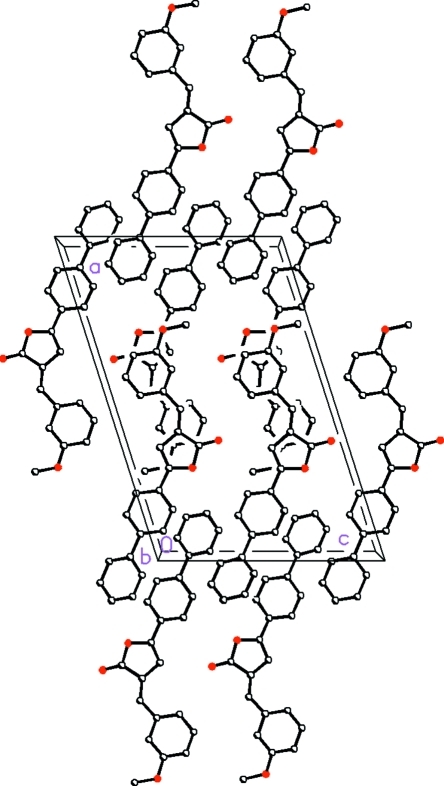
Packing diagram of the title compound, viewed down the b axis.

## supplementary crystallographic information

### Comment

Certain drugs containing a furanone structure were potential anti-ulcer agents because they did not irritate the lining of the stomach (Felman *et al.*, 1992). Their occurrence in nature has been exploited in the pharmaceutical industry because of their unusual biological activities, such as anti-ulcer and anti-cancer treatments. The antitumor activity of several analogs of furanones was evaluated using both in vivo and in vitro methods on mice, where oral adminstration showed a relative decrease in tumor growth. In an effort to create more efficient drugs, scientists began to explore the role of furanones in the biochemical processes of the human body (Rappai *et al.*, 2009). The gastrointestinal toxicity of acidic non-steroidal anti- inflammatory drugs (NSAIDs) is one of the most challenging problems in medicinal chemistry (Husain *et al.*, 2010). NSAIDs form a class of therapeutic agents that are most widely used world over because of their antiinflammatory, analgesic and antipyretic effects. Aroylpropionic acids and furanones are effective anti-inflammatory agents and some of them are available in the market, however, they are associated with gastrointestinal side effects; a common feature of NSAIDs (Cioli *et al.*, 1979). Studies suggest that the direct tissue contact of these agents plays an important role in the production of side effects and the reported literature confirms that gastrointestinal side effects of aroylpropionic acids are due to the presence of the free carboxylic group in the parent drug. This free carboxylic group, therefore, has been converted to the furanone ring to get a compound free from GIT side effects. Furanones and b\-aroylpropionic acids are important intermediates in heterocyclic chemistry and have been used for the synthesis of various biologically active five-membered heterocyles such as butenolides, pyrrolones (Husain *et al.*, 2005; Khan *et al.*, 2002) oxadiazoles (Husain *et al.*, 2009) and triazoles (Hashem *et al.*, 2007). The crystal structure study of a related compound, (E)-6-methoxy-3-( -methoxybenzylidene)benzo[b]furan-2(3H)-one, at 173 K is reported (Burke *et al.*, 2000). In view of the importance of the title compound, (I), this paper reports its crystal structure.

In the title molecule, the dihedral angles between the mean planes of the five-menbered furan ring and the methoxy substituted benzene and biphenyl benzene rings are 2.43 (7)°, 4.48 (7)°, and 30.47 (8)°, respectively (Fig. 1).  Bond lengths are in normal ranges (Allen *et al.*, 1987).  The crystal packing is stabiized by weak C—H···O and C—H···π intermolecular hydrogen bonds and π-π stacking (Table 2) interactions (Fig. 2).

### Experimental

A solution of 3-(4-phenylbenzoyl)propionic acid (0.71 g, 3 mmol) and 3-methoxybenzaldehyde (0.45 g, 3 mmol) in a acetic anhydride (5 ml) with triethylamine (3–4 drops) was refluxed for 5–6 hrs on a water bath under anhydrous conditions. After completion of the reaction, the contents were poured into crushed ice in small portions while stirring. A solid mass separated out, which was filtered, washed with water and crystallized from 2-butanone to get X-ray quality crystals (m.p. 411–413 K).

### Refinement

All of the H atoms were placed in their calculated positions and then refined using the riding model with C–H lengths of 0.95 Å (CH) or 0.98 Å (CH_3_). The isotropic displacement parameters for these atoms were set to 1.18–1.21 (CH) or 1.49 (CH_3_) times *U*_eq_ of the parent atom.

### Crystal data

**Table:**

C_24_H_18_O_3_	*F*(000) = 744
*M**_r_* = 354.38	*D*_x_ = 1.341 Mg m^−^^3^
Monoclinic, *P*2_1_/*c*	Mo *K*α radiation, λ = 0.71073 Å
Hall symbol: -P 2ybc	Cell parameters from 5822 reflections
*a* = 19.8766 (8) Å	θ = 3.1–32.2°
*b* = 6.9914 (3) Å	µ = 0.09 mm^−^^1^
*c* = 13.2603 (6) Å	*T* = 170 K
β = 107.735 (4)°	Block, pale yellow
*V* = 1755.15 (13) Å^3^	0.22 × 0.22 × 0.12 mm
*Z* = 4	

### Data collection

**Table:**

Oxford Diffraction Xcalibur Eos Gemini diffractometer	4534 independent reflections
Radiation source: Enhance (Mo) X-ray Source	3310 reflections with *I* > 2σ(*I*)
graphite	*R*_int_ = 0.029
Detector resolution: 16.1500 pixels mm^-1^	θ_max_ = 28.7°, θ_min_ = 3.2°
ω scans	*h* = −26→26
Absorption correction: multi-scan (*CrysAlis RED*; Oxford Diffraction, 2010)	*k* = −9→9
*T*_min_ = 0.981, *T*_max_ = 0.990	*l* = −17→17
19756 measured reflections	

### Refinement

**Table:**

Refinement on *F*^2^	Primary atom site location: structure-invariant direct methods
Least-squares matrix: full	Secondary atom site location: difference Fourier map
*R*[*F*^2^ > 2σ(*F*^2^)] = 0.048	Hydrogen site location: inferred from neighbouring sites
*wR*(*F*^2^) = 0.143	H-atom parameters constrained
*S* = 1.01	*w* = 1/[σ^2^(*F*_o_^2^) + (0.0722*P*)^2^ + 0.299*P*] where *P* = (*F*_o_^2^ + 2*F*_c_^2^)/3
4534 reflections	(Δ/σ)_max_ = 0.001
245 parameters	Δρ_max_ = 0.23 e Å^−^^3^
0 restraints	Δρ_min_ = −0.16 e Å^−^^3^

### Special details

**Table:**

Geometry. All e.s.d.'s (except the e.s.d. in the dihedral angle between two l.s. planes) are estimated using the full covariance matrix. The cell e.s.d.'s are taken into account individually in the estimation of e.s.d.'s in distances, angles and torsion angles; correlations between e.s.d.'s in cell parameters are only used when they are defined by crystal symmetry. An approximate (isotropic) treatment of cell e.s.d.'s is used for estimating e.s.d.'s involving l.s. planes.
Refinement. Refinement of *F*^2^ against ALL reflections. The weighted *R*-factor *wR* and goodness of fit *S* are based on *F*^2^, conventional *R*-factors *R* are based on *F*, with *F* set to zero for negative *F*^2^. The threshold expression of *F*^2^ > σ(*F*^2^) is used only for calculating *R*-factors(gt) *etc*. and is not relevant to the choice of reflections for refinement. *R*-factors based on *F*^2^ are statistically about twice as large as those based on *F*, and *R*- factors based on ALL data will be even larger.

### Fractional atomic coordinates and isotropic or equivalent isotropic displacement parameters (Å^2^)

**Table:**

	*x*	*y*	*z*	*U*_iso_*/*U*_eq_	
O1	0.71593 (5)	0.14726 (16)	0.22178 (7)	0.0415 (3)	
O2	0.63009 (6)	0.13953 (19)	0.06638 (8)	0.0539 (3)	
O3	0.28081 (5)	0.15321 (18)	0.15671 (9)	0.0516 (3)	
C1	1.04917 (7)	0.3141 (2)	0.59764 (12)	0.0393 (3)	
H1A	1.0398	0.3604	0.5274	0.047*	
C2	1.11564 (8)	0.3377 (2)	0.66876 (14)	0.0470 (4)	
H2A	1.1514	0.4004	0.6473	0.056*	
C3	1.13016 (8)	0.2703 (3)	0.77084 (13)	0.0497 (4)	
H3A	1.1759	0.2862	0.8199	0.060*	
C4	1.07787 (9)	0.1797 (3)	0.80145 (13)	0.0500 (4)	
H4A	1.0879	0.1325	0.8717	0.060*	
C5	1.01101 (8)	0.1571 (2)	0.73078 (12)	0.0411 (3)	
H5A	0.9753	0.0958	0.7531	0.049*	
C6	0.99552 (7)	0.2235 (2)	0.62704 (11)	0.0330 (3)	
C7	0.92411 (7)	0.19992 (19)	0.55035 (10)	0.0317 (3)	
C8	0.86347 (7)	0.2076 (2)	0.58311 (11)	0.0395 (3)	
H8A	0.8685	0.2235	0.6562	0.047*	
C9	0.79673 (7)	0.1927 (2)	0.51171 (11)	0.0389 (3)	
H9A	0.7565	0.1986	0.5361	0.047*	
C10	0.78789 (7)	0.16882 (19)	0.40409 (10)	0.0315 (3)	
C11	0.84804 (7)	0.1561 (2)	0.37113 (11)	0.0365 (3)	
H11A	0.8431	0.1359	0.2984	0.044*	
C12	0.91467 (7)	0.1724 (2)	0.44324 (11)	0.0363 (3)	
H12A	0.9549	0.1646	0.4190	0.044*	
C13	0.71770 (7)	0.1588 (2)	0.32790 (10)	0.0329 (3)	
C14	0.65258 (7)	0.16084 (19)	0.33828 (10)	0.0331 (3)	
H14A	0.6416	0.1683	0.4030	0.040*	
C15	0.60244 (7)	0.14966 (19)	0.23409 (10)	0.0322 (3)	
C16	0.64589 (7)	0.1442 (2)	0.16115 (11)	0.0379 (3)	
C17	0.53146 (7)	0.1432 (2)	0.19272 (10)	0.0340 (3)	
H17A	0.5149	0.1351	0.1177	0.041*	
C18	0.47600 (7)	0.14655 (19)	0.24314 (10)	0.0318 (3)	
C19	0.40626 (7)	0.1452 (2)	0.17584 (11)	0.0340 (3)	
H19A	0.3975	0.1408	0.1013	0.041*	
C20	0.35005 (7)	0.1504 (2)	0.21690 (11)	0.0363 (3)	
C21	0.36229 (8)	0.1503 (2)	0.32559 (12)	0.0418 (4)	
H21A	0.3238	0.1504	0.3540	0.050*	
C22	0.43071 (8)	0.1501 (2)	0.39204 (12)	0.0436 (4)	
H22A	0.4390	0.1498	0.4665	0.052*	
C23	0.48761 (7)	0.1505 (2)	0.35246 (11)	0.0383 (3)	
H23A	0.5344	0.1534	0.3995	0.046*	
C24	0.26584 (9)	0.1751 (3)	0.04562 (13)	0.0543 (4)	
H24A	0.2147	0.1852	0.0125	0.081*	
H24B	0.2838	0.0640	0.0168	0.081*	
H24C	0.2888	0.2913	0.0309	0.081*	

### Atomic displacement parameters (Å^2^)

**Table:**

	*U*^11^	*U*^22^	*U*^33^	*U*^12^	*U*^13^	*U*^23^
O1	0.0317 (5)	0.0641 (7)	0.0309 (5)	0.0013 (5)	0.0128 (4)	0.0002 (4)
O2	0.0438 (6)	0.0898 (9)	0.0293 (5)	0.0029 (6)	0.0128 (5)	0.0027 (5)
O3	0.0273 (5)	0.0822 (9)	0.0436 (6)	0.0008 (5)	0.0084 (4)	0.0029 (6)
C1	0.0339 (7)	0.0437 (8)	0.0425 (8)	0.0023 (6)	0.0150 (6)	0.0029 (6)
C2	0.0317 (7)	0.0508 (9)	0.0608 (10)	−0.0004 (6)	0.0174 (7)	−0.0006 (8)
C3	0.0302 (7)	0.0584 (10)	0.0535 (10)	0.0033 (7)	0.0026 (7)	−0.0021 (8)
C4	0.0437 (8)	0.0595 (11)	0.0415 (8)	0.0041 (7)	0.0048 (7)	0.0051 (7)
C5	0.0378 (7)	0.0458 (9)	0.0401 (8)	−0.0024 (6)	0.0121 (6)	0.0039 (6)
C6	0.0296 (6)	0.0338 (7)	0.0364 (7)	0.0029 (5)	0.0110 (5)	−0.0006 (5)
C7	0.0293 (6)	0.0323 (7)	0.0342 (7)	0.0012 (5)	0.0110 (5)	0.0009 (5)
C8	0.0340 (7)	0.0550 (9)	0.0312 (7)	−0.0010 (6)	0.0125 (6)	−0.0033 (6)
C9	0.0300 (7)	0.0542 (9)	0.0360 (7)	−0.0004 (6)	0.0150 (6)	−0.0035 (6)
C10	0.0298 (6)	0.0325 (7)	0.0334 (7)	0.0005 (5)	0.0114 (5)	0.0007 (5)
C11	0.0362 (7)	0.0453 (8)	0.0301 (7)	0.0011 (6)	0.0133 (5)	−0.0012 (6)
C12	0.0305 (6)	0.0445 (8)	0.0379 (7)	0.0015 (6)	0.0166 (6)	−0.0010 (6)
C13	0.0343 (7)	0.0370 (7)	0.0281 (6)	0.0008 (5)	0.0106 (5)	−0.0007 (5)
C14	0.0305 (6)	0.0393 (7)	0.0302 (7)	0.0004 (5)	0.0102 (5)	−0.0002 (5)
C15	0.0334 (7)	0.0345 (7)	0.0298 (6)	0.0028 (5)	0.0115 (5)	0.0018 (5)
C16	0.0333 (7)	0.0504 (9)	0.0307 (7)	0.0027 (6)	0.0108 (6)	0.0024 (6)
C17	0.0328 (7)	0.0409 (8)	0.0279 (6)	0.0027 (6)	0.0088 (5)	0.0030 (5)
C18	0.0311 (6)	0.0332 (7)	0.0310 (6)	0.0011 (5)	0.0091 (5)	0.0005 (5)
C19	0.0327 (7)	0.0397 (8)	0.0289 (6)	0.0001 (5)	0.0085 (5)	−0.0001 (5)
C20	0.0294 (6)	0.0403 (8)	0.0379 (7)	−0.0007 (5)	0.0086 (6)	−0.0009 (6)
C21	0.0375 (7)	0.0530 (9)	0.0401 (8)	0.0002 (6)	0.0196 (6)	−0.0016 (7)
C22	0.0454 (8)	0.0560 (10)	0.0309 (7)	0.0006 (7)	0.0140 (6)	−0.0016 (6)
C23	0.0323 (7)	0.0486 (8)	0.0320 (7)	0.0003 (6)	0.0067 (5)	0.0003 (6)
C24	0.0369 (8)	0.0779 (13)	0.0418 (9)	0.0006 (8)	0.0027 (7)	−0.0008 (8)

### Geometric parameters (Å, °)

**Table:**

O1—C16	1.3795 (16)	C10—C13	1.4537 (18)
O1—C13	1.3991 (15)	C11—C12	1.3819 (19)
O2—C16	1.1994 (16)	C11—H11A	0.95
O3—C20	1.3647 (16)	C12—H12A	0.95
O3—C24	1.4195 (19)	C13—C14	1.3427 (18)
C1—C2	1.379 (2)	C14—C15	1.4385 (18)
C1—C6	1.3941 (19)	C14—H14A	0.95
C1—H1A	0.95	C15—C17	1.3499 (18)
C2—C3	1.378 (2)	C15—C16	1.4803 (19)
C2—H2A	0.95	C17—C18	1.4531 (18)
C3—C4	1.380 (2)	C17—H17A	0.95
C3—H3A	0.95	C18—C23	1.3971 (19)
C4—C5	1.382 (2)	C18—C19	1.4016 (18)
C4—H4A	0.95	C19—C20	1.3845 (19)
C5—C6	1.3945 (19)	C19—H19A	0.95
C5—H5A	0.95	C20—C21	1.387 (2)
C6—C7	1.4821 (18)	C21—C22	1.377 (2)
C7—C12	1.3882 (18)	C21—H21A	0.95
C7—C8	1.4010 (18)	C22—C23	1.384 (2)
C8—C9	1.3792 (19)	C22—H22A	0.95
C8—H8A	0.95	C23—H23A	0.95
C9—C10	1.3937 (19)	C24—H24A	0.98
C9—H9A	0.95	C24—H24B	0.98
C10—C11	1.3954 (18)	C24—H24C	0.98
			
C16—O1—C13	107.39 (10)	C14—C13—C10	132.78 (12)
C20—O3—C24	117.63 (12)	O1—C13—C10	115.25 (11)
C2—C1—C6	121.21 (14)	C13—C14—C15	107.96 (12)
C2—C1—H1A	119.4	C13—C14—H14A	126.0
C6—C1—H1A	119.4	C15—C14—H14A	126.0
C3—C2—C1	120.07 (15)	C17—C15—C14	136.44 (13)
C3—C2—H2A	120.0	C17—C15—C16	118.63 (12)
C1—C2—H2A	120.0	C14—C15—C16	104.92 (11)
C2—C3—C4	119.65 (14)	O2—C16—O1	120.44 (12)
C2—C3—H3A	120.2	O2—C16—C15	131.81 (13)
C4—C3—H3A	120.2	O1—C16—C15	107.74 (11)
C3—C4—C5	120.52 (15)	C15—C17—C18	131.15 (13)
C3—C4—H4A	119.7	C15—C17—H17A	114.4
C5—C4—H4A	119.7	C18—C17—H17A	114.4
C4—C5—C6	120.57 (14)	C23—C18—C19	118.66 (12)
C4—C5—H5A	119.7	C23—C18—C17	124.69 (12)
C6—C5—H5A	119.7	C19—C18—C17	116.66 (12)
C1—C6—C5	117.98 (12)	C20—C19—C18	120.63 (12)
C1—C6—C7	120.87 (12)	C20—C19—H19A	119.7
C5—C6—C7	121.16 (12)	C18—C19—H19A	119.7
C12—C7—C8	117.49 (12)	O3—C20—C19	124.14 (13)
C12—C7—C6	121.34 (12)	O3—C20—C21	115.74 (12)
C8—C7—C6	121.16 (12)	C19—C20—C21	120.11 (13)
C9—C8—C7	121.52 (13)	C22—C21—C20	119.45 (13)
C9—C8—H8A	119.2	C22—C21—H21A	120.3
C7—C8—H8A	119.2	C20—C21—H21A	120.3
C8—C9—C10	120.45 (13)	C21—C22—C23	121.27 (13)
C8—C9—H9A	119.8	C21—C22—H22A	119.4
C10—C9—H9A	119.8	C23—C22—H22A	119.4
C9—C10—C11	118.40 (12)	C22—C23—C18	119.84 (13)
C9—C10—C13	120.82 (12)	C22—C23—H23A	120.1
C11—C10—C13	120.78 (12)	C18—C23—H23A	120.1
C12—C11—C10	120.66 (13)	O3—C24—H24A	109.5
C12—C11—H11A	119.7	O3—C24—H24B	109.5
C10—C11—H11A	119.7	H24A—C24—H24B	109.5
C11—C12—C7	121.45 (12)	O3—C24—H24C	109.5
C11—C12—H12A	119.3	H24A—C24—H24C	109.5
C7—C12—H12A	119.3	H24B—C24—H24C	109.5
C14—C13—O1	111.96 (11)		
			
C6—C1—C2—C3	0.4 (2)	C11—C10—C13—O1	−3.56 (19)
C1—C2—C3—C4	−0.1 (3)	O1—C13—C14—C15	0.14 (16)
C2—C3—C4—C5	−0.4 (3)	C10—C13—C14—C15	179.15 (14)
C3—C4—C5—C6	0.7 (2)	C13—C14—C15—C17	178.84 (16)
C2—C1—C6—C5	0.0 (2)	C13—C14—C15—C16	−0.94 (15)
C2—C1—C6—C7	179.58 (13)	C13—O1—C16—O2	178.09 (14)
C4—C5—C6—C1	−0.5 (2)	C13—O1—C16—C15	−1.35 (15)
C4—C5—C6—C7	179.88 (14)	C17—C15—C16—O2	2.2 (2)
C1—C6—C7—C12	33.2 (2)	C14—C15—C16—O2	−177.94 (17)
C5—C6—C7—C12	−147.21 (15)	C17—C15—C16—O1	−178.42 (12)
C1—C6—C7—C8	−145.78 (15)	C14—C15—C16—O1	1.42 (15)
C5—C6—C7—C8	33.8 (2)	C14—C15—C17—C18	0.4 (3)
C12—C7—C8—C9	−1.4 (2)	C16—C15—C17—C18	−179.83 (13)
C6—C7—C8—C9	177.64 (13)	C15—C17—C18—C23	−2.6 (2)
C7—C8—C9—C10	0.2 (2)	C15—C17—C18—C19	177.53 (14)
C8—C9—C10—C11	1.5 (2)	C23—C18—C19—C20	0.9 (2)
C8—C9—C10—C13	−178.10 (13)	C17—C18—C19—C20	−179.26 (13)
C9—C10—C11—C12	−1.9 (2)	C24—O3—C20—C19	−7.8 (2)
C13—C10—C11—C12	177.69 (13)	C24—O3—C20—C21	173.06 (14)
C10—C11—C12—C7	0.7 (2)	C18—C19—C20—O3	178.70 (13)
C8—C7—C12—C11	1.0 (2)	C18—C19—C20—C21	−2.2 (2)
C6—C7—C12—C11	−178.05 (13)	O3—C20—C21—C22	−179.16 (13)
C16—O1—C13—C14	0.79 (16)	C19—C20—C21—C22	1.7 (2)
C16—O1—C13—C10	−178.41 (11)	C20—C21—C22—C23	0.2 (2)
C9—C10—C13—C14	−3.0 (2)	C21—C22—C23—C18	−1.5 (2)
C11—C10—C13—C14	177.46 (15)	C19—C18—C23—C22	1.0 (2)
C9—C10—C13—O1	176.00 (12)	C17—C18—C23—C22	−178.89 (14)

### Hydrogen-bond geometry (Å, °)

**Table:**

Cg1 is the centroid of the C1–C6 ring.

**Table:**

*D*—H···*A*	*D*—H	H···*A*	*D*···*A*	*D*—H···*A*
C2—H2A···O3^i^	0.95	2.56	3.3358 (18)	138
C5—H5A···Cg1^ii^	0.95	2.69	3.4762 (16)	141

Symmetry codes: (i) *x*+1, −*y*+1/2, *z*+1/2; (ii) −*x*+2, *y*−1/2, −*z*+3/2.

### Table 2 π-π-stacking interactions.

**Table:**

*Cg*I···*Cg*J	CgI···CgJ (Å)	CgI···Perp (Å)	CgJ···Perp (Å)
Cg2···Cg3^i^	3.8752 (8)	3.5136 (6)	-3.4926 (6)
Cg2···Cg3^ii^	3.8331 (8)	-3.4772 (6)	3.4889 (6)

Cg2 and Cg3 are the centroids of rings O1/C13–C16 and C18–C23, respectively.Symmetry codes: (i) 1-x, -1/2+y, 1/2-z; (ii) 1-x, 1/2+y, 1/2-z.
